# Mycoplasma Pneumoniae and Chlamydia Pneumoniae Seropositivity in Patients With Age-Related Macular Degeneration

**DOI:** 10.4021/jocmr2010.03.282w

**Published:** 2010-03-31

**Authors:** Burak Turgut, Fatma Uyar, Fulya Ilhan, Tamer Demir, Ulku Celiker

**Affiliations:** a Firat University School of Medicine, Department of Ophthalmology, Elazig, Turkey; b Firat University School of Medicine, Department of Immunology, Elazig, Turkey

## Abstract

**Background:**

To determine a possible relation between Mycoplasma pneumoniae (MP) or Chlamidia pneumoniae (CP) seropositivity and age-related macular degeneration (AMD).

**Methods:**

Sixty patients (20 wet AMD, 20 dry AMD and 20 non-AMD controls) were included in the study. Serum samples were collected for analysis of IgM and IgG antibody seropositivity for CP and MP by enzyme-linked immunosorbent assay (ELISA). Comparison of the distribution of seropositivity of these antibodies among patients with wet and dry AMD, and controls was performed. A prospective comparative clinical trial was applied.

**Results:**

There was no major difference in the distribution of IgM and IgG seropositivity to CP and MP in patients with wet and dry AMD, and in controls (p > 0.05).

**Conclusions:**

We found no significant association between MP as well as CP antibody titers and AMD. It seems that MP or CP infection is not a risk factor for AMD.

**Keywords:**

Mycoplasma pneumoniae; Chlamydia pneumoniae; Age-related macular degeneration; Serology

## Introduction

Age-related macular degeneration (AMD) is a major cause of irreversible visual loss in developed countries in 65 years and older. Although the pathogenesis of AMD is not clearly understood, it has been considered that AMD is multifactorial disease. Risk factors associated with AMD include smoking, diet, light exposure, advanced age, race, family history (genetics), body mass index, underlying cardiovascular disease, and systemic inflammatory or infectious diseases [1-3]. A number of clinical and experimental studies also suggest a role for inflammation or infection. The potential role of infectious agents as a trigger for inflammation in the pathogenesis of AMD as well as systemic vascular diseases is a subject of continuing debate [4-6].

In recent studies, Cytomegalovirus (CMV), Chlamydia pneumoniae (CP), and Helicobacter pylori (HP) have been linked to AMD. The link between CP and AMD is still controversial. Kalayoglu et al have demonstrated the presence of CP in choroidal neovascular membranes (CNVM) related with AMD and the serological association between CP infection and AMD [7, 8] whilst in two recent studies, Robman et al and Kessler et al failed to show an association between CP and AMD or AMD CNVM, respectively [9, 10]. There is no data in literature concerning the association between AMD pathogenesis and Mycoplasma pneumonia (MP), another atypical bacterium.

Mycoplasmas, which are the smallest and simplest self-replicating microorganisms, can exist as a persistent asymptomatic infection, resulting in chronic inflammation as well as CP [11, 12]. MP is a significant respiratory pathogen in persons of all ages, causing respiratory disease, and it may induce clinically significant manifestations in extra-pulmonary sites by direct invasion and/or immunologic effects. Macrophage activation, cytokine induction, and super-antigen properties are some factors related to the pathogenity of Mycoplasmas [11-14]. In recent studies, the association of MP and CP has been described in pneumonia, astma, aortic stenosis, coronary atherosclerosis, and stroke or in immunodeficient patients [15-20]. In addition, it is considered that co-infections with CP in association with MP potentiate the virulence of both agents. Therefore, when circulating antibodies of both these two agents are present in patients with AMD, they may increase each others pathogenicity [21, 22].

Therefore, we hypothesized that infection or postinfection with MP may be associated with AMD and we purposed to determine this possible association.

## Patients and Methods

This pilot study was designed as a prospective case-control study and 20 patients with dry AMD (Group 1), 20 patients with wet AMD (Group 2) and 20 subjects age- and sex-matched and without AMD (Group 3) were included in the study. All patients underwent a complete ophthalmologic and general examination. The protocol for the study was reviewed and approved by the institutional review board. The tenets of the Helsinki declaration were followed throughout the study. Informed consent was obtained from each subject including detailed explanation of all procedures before participation in the study. A staff retina specialist classified AMD into nonneovascular or neovascular stages of disease.

### Inclusion criteria

In group 1, the patients with dry (nonneovascular) AMD characterized by macular drusen or the presence of geographic atrophy without choroidal neovascularization (NV) or scarring documented by Color fundus imaging, Fundus Fluorescein Angiography (FFA) and Optic Coherence Tomography (OCT).

In group 2, the patients with wet AMD with CNVM or disciform scar documented by FFA and OCT.

In group 3, control subjects that are sex- and age-matched subjects included patients presenting for routine eye examination with or without other ocular disorders, but without evidence of drusen, retina pigment epithelial changes or choroidal neovascularization.

The participants with diabetes mellitus, ocular or systemic infection and inflammation, any hematological and immune disease, malignancy, hypergammaglobulinemia, connective tissue disease, history of myocardial infarction and coronary artery disease, or history of the usage of the drug that could influence serum immunoglobulin levels and previous laser treatment or intravitreal injection were excluded from the study.

### Serological assay

Peripheral venous blood (10 ml) was collected from patients and healthy controls by venipuncture. The serum was separated and stored frozen at -20^o^C. Samples were encoded, and laboratory personnel were masked to clinical information on the patients. Serological analysis as described in the following subsections was made by same personnel.

Qualitative immunoenzymatic determination of human CP IgG and IgM in serum were made using Novatec/Immunodiagnostica GmbH Germany (product No: CHLM0070, CHLG0070) on automatised ELISA system. Microtiter strip wells are precoated with CP antigens to bind corresponding antibodies of the specimen. After washing the wells to remove all unbound sample material horseradish peroxidase (HRP) labeled anti-human IgG (IgM) conjugate is added. This conjugate binds to the captured Chlamydia specific antibodies. The immune complex formed by the bound conjugate is visualized by adding tetrametilbenzidine substrate which gives a blue reaction product. The intensity of this product is proportional to the amount of Chlamydia specific IgG (IgM) antibodies in the specimen. Sulphuric acid is added to stop the reaction. This produces a yellow endpoint color. Absorbance at 450 nm infrared was read using an ELISA micro well plate reader.

The absorbance of all wells at 450 nm was measured and the absorbance values for each control and patients samples was recorded for identification absorbance distribution. The cut-off value was calculated according to absorbance value. Samples were considered positive if the absorbance value is higher than 10% over the cut-off. All results were given as a NovaTec Units (NTU) (Cut-off:10 NTU).

Quantitative tests for detection of human antibodies IgG and IgM in serum against MP (Virion/Serion GmbH Germany (product No: ESR127G and ESR127M) ELISA kit.

Antibodies activities were given in U/ml. For MP IgG calculation was interpreted positive results >30 U/ml, borderline results 20 - 30 U/ml and negative results < 20 U/ml, and for IgM was positive results >17 U/ml, borderline results 13 - 17 U/ml, negative results <13 U/ml. All ELISA reactions were performed according to manufacturers instructions on Dynex 1DXC-1381 (USA) full automatic ELISA system.

### Statistical analysis

Statistical analysis was performed using the Statistical Package for the Social Sciences version 13.0 (SPSS, Inc., Chicago, IL, USA). The Student t test or chi-square test was used to compare nominal and categorical variables among the study groups, respectively. Results were given as means ± standard deviations. P value less than 0.05 was considered as statistically significant.

## Results

The subjects included 13 men (65%) and 7 women (35%) in Group 1; 12 men (60%) and 8 women (40%) in Group 2; 10 men (50%) and 10 women (50%) in Group 3. The mean ages of the Group 1, Group 2 and Group 3 were 68.9 ± 4.99 years (range, 56 to 78 years), 65.85 ± 4.91 years (range, 58 to 74 years), 66.50 ± 4.84 years (range, 55 to 80 years), respectively. The patient and control groups were matched for age and sex and there was no statistically significant difference with regard to age and gender among the groups (p > 0.05). The demographical data in the study groups are shown in Table 1.

**Table 1:** Demographical Data in the Study Groups

Group (n)	Mean Age (range)	Gender (%)
Dry AMD (20)	68.90 ± 4.99 years (56 - 78)	13 men (65%), 7 women (35%)
Wet AMD (20)	65.85 ± 4.91 years (58 - 74)	12 men (60%), 8 women (40%)
Non-AMD controls (20)	66.50 ± 4.84 years (55 - 80)	10 men (50%), 10 women (50%)

None of 60 patients in the study groups had positive for Ig M antibody titers to CP. Two (10%) of 20 patients with wet AMD had positive for IgG antibody titers to CP, compared with none of 20 patients with dry AMD and none of 20 controls. There was no major difference in the distribution of IgM and IgG titers for CP in wet and dry AMD (p > 0.05).

Similarly, there was no major difference in the distribution of IgM and IgG titers for MP in wet and dry AMD, and in controls (p > 0.05). All of the patients with wet AMD and dry AMD had negative for IgM antibody titers to MP whilst 19 (95%) of 20 controls had negative. Borderline IgM titers to MP were only one in the controls. One (5%) of 20 patients with wet AMD had positive for IgG antibody titers to MP, compared with two (10%) of 20 dry AMD patients. One (5%) of 20 controls had positive. Borderline IgG titers to MP were observed in three patients (15%) in wet AMD group but only one (5%) in each of dry AMD and control group. IgM and IgG seropositivities to CP and MP in the study groups are shown in Table 2.

**Table 2:** IgM and IgG Seropositivities to CP and MP (%)

Group	CP IgM Seropositivity (%)	CP IgG Seropositivity (%)	MP IgM Seropositivity (%)	MP IgG Seropositivity (%)
Group 1 (n = 20)	Positive: 0 (0%) Negative: 20 (100%)	Positive: 0 (0%) Negative: 20 (100%)	Positive: 0 (0%) Borderline: 0 (0%) Negative: 20 (100%)	Positive: 2 (10%) Borderline: 1(5%) Negative: 17 (85%)
Group 2 (n = 20)	Positive: 0 (0%) Negative: 20 (100%)	Positive: 2 (10%) Negative: 18 (90%)	Positive: 0 (0%) Borderline: 0 (0%) Negative: 20 (100%)	Positive: 1 (5%) Borderline: 3(15%) Negative: 16(80%)
Group 3 (n = 20)	Positive: 0 (0%) Negative: 20 (100%)	Positive: 0 (0%) Negative: 20 (100%)	Positive: 0 (0%) Borderline: 1 (5%) Negative: 19 (95%)	Positive: 1 (5%) Borderline: 1(5%) Negative: 18(90%)

The mean serum IgM titers for CP in patients with dry AMD (Group 1), wet AMD (Group 2) and in the controls (Group 3) were 2.09 ± 1.56, 1.27 ± 1.18 and 1.48 ± 0.53 NTU/mL, respectively. The mean serum IgG titers for CP in patients with dry AMD (Group 1), wet AMD (Group 2) and in the controls (Group 3) were 5± 4.72, 4.29 ± 3.51 and 3.30 ± 2.06 NTU/mL, respectively. There was no significant difference among the serum IgM and IgG titers of the study groups (p > 0.05).

The mean serum IgM titers for MP in patients with dry AMD (Group 1), wet AMD (Group 2) and in the controls (Group 3) were 3.13 ± 1.03, 4.13 ± 1.99 and 4.13 ± 2.63 mlU/mL, respectively. The mean serum IgG titers for MP in patients with dry AMD (Group 1), wet AMD (Group 2) and in the controls (Group 3) were 9.95 ± 9.89, 12.99 ± 8.75 and 15.78 ± 8.32 U/mL, respectively. There was no significant difference among the serum IgM and IgG titers of the study groups (p > 0.05).

## Discussion

A number of studies in humans and animals suggest that inflammation or infection may play a role in the pathogenesis of AMD. There is some evidence the relation between AMD and infections due to CMV, HP or CP [7-10, 23, 24]. Kalayoglu and Robman and their coworkers demonstrated that serum antibodies for CP proteins are associated with increased risk for the development and progression of AMD [7, 10]. In another study, Kalayoglu et al. demonstrated the presence of CP in AMD CNVM when compared to the eyes with AMD without CNVM and non-AMD eyes [8]. They reported that macrophages may produce VEGF after infection with CP. In addition, Miller et al investigated the possible association between AMD and CP, HP, and CMV, as these agents are known to cause chronic infection and inflammation and CMV has been shown in animal models to accelerate the development of atherosclerosis. Miller et al found a significant association between high anti-CMV IgG titers and wet AMD compared with control and dry AMD patients. However, they did not find a difference in the distribution of titers for CP and HP in wet and dry AMD patients. They proposed that CMV-infected macrophages and endothelial cells might release increased amounts of proinflammatory cytokines that could cause the development of CNVM [23].

In another study, Ishida et al also found significantly elevated anti-CP IgG antibodies in patients with wet AMD compared with control subjects [24]. In contrast to these studies, two recent studies by Robman et al and Kessler et al failed to show an association between CP and AMD or AMD CNV, respectively [9, 10].

In our study, we expected elevation in CP antibodies in patients with specially wet AMD. However, distributions of CP antibodies were not found to be significantly different between patients with AMD and controls. These findings are contrary to those in the previous reports which demonstrated an association between CP infection and AMD. Our data supports the controversial role of CP in AMD pathogenesis.

To the best of our knowledge, this is the first report investigating the possible association between MP and AMD. In present study, we could not find a relation between AMD and MP. The insignificant results concerning with serology of MP in our study may be due to local inflammation. Thus, the histopathological or immunohistochemical studies in AMD CNVMs would be providing to understand the possible role of MP in AMD pathogenesis.

Although sample size of our study may be a limitation in this study, sufficient data to support this relationship could not be obtained. But it seems that MP or CP infection is not be related with the development of AMD. The long-term and larger studies in the series will contribute to verification of this relationship.
